# Deletion of Long Isoform of Eukaryotic Elongation Factor 1Bδ Leads to Audiogenic Seizures and Aversive Stimulus-Induced Long-Lasting Activity Suppression in Mice

**DOI:** 10.3389/fnmol.2018.00358

**Published:** 2018-10-02

**Authors:** Taku Kaitsuka, Hiroshi Kiyonari, Aki Shiraishi, Kazuhito Tomizawa, Masayuki Matsushita

**Affiliations:** ^1^Department of Molecular Physiology, Faculty of Life Sciences, Kumamoto University, Kumamoto, Japan; ^2^Animal Resource Development Unit, RIKEN Center for Life Science Technologies, Kobe, Japan; ^3^Genetic Engineering Team, RIKEN Center for Life Science Technologies, Kobe, Japan; ^4^Department of Molecular and Cellular Physiology, Graduate School of Medicine, University of the Ryukyus, Okinawa, Japan

**Keywords:** elongation factor, alternative splicing, audiogenic seizure, fear conditioning, protein synthesis

## Abstract

Alternative splicing enables a gene to give rise to diverse protein products. The *Eef1d* gene produces two isoforms: a short isoform that encodes translation elongation factor 1Bδ (eEF1Bδ1), and a long isoform that encodes the heat shock-responsive transcription factor eEF1BδL. Previously, we found that eEF1BδL was a splice variant that was specific to the brain and testis, and the protein encoded is thought to have a function in the central nervous system. In this study, we generated knockout (KO) mice of C57BL/6J background that selectively lacked a specific exon in *Eef1d* for the long isoform. These KO mice lacked eEF1BδL, but not eEF1Bδ1, in the brain. Although the KO mice showed normal anxiety-related and learning behavior in behavioral tests, some showed severe seizures in response to loud sounds (90 dBA), an audiogenic seizures (AGS) response. Furthermore, after the KO mice had been subjected to the fear conditioning test, they showed remarkably decreased locomotor activity in their home cage and in the open-field and elevated plus-maze tests. After the fear conditioning test, a significant decrease in brain weight, atrophy of the hippocampus and midbrain, and reduced cortical layer thickness were observed in the KO mice. We also found a compensatory increase in the eEF1Bδ1 level and elevated protein synthesis with the induction of endoplasmic reticulum stress markers in these mice. Our results suggest that eEF1BδL has an important role in normal brain function especially when exposed to external stimuli.

## Introduction

Alternative pre-mRNA splicing can result in mRNA isoforms that encode distinct protein products, which may differ in structure, function, localization or other properties (Stamm et al., 2005; Wang et al., 2008; Licatalosi and Darnell, 2010). Tissue- and development-specific splicing and the resulting protein isoforms have a critical role in normal development in mammals and dysregulation of alternative splicing is the cause of many human diseases (Chen and Manley, 2009; Kalsotra and Cooper, 2011; Scotti and Swanson, 2016). In the brain, alternative splicing regulates many functions as neurogenesis, neuronal migration, synaptogenesis, cell survival and synaptic functions during development and in mature neurons (Ule and Darnell, 2006; Vuong et al., 2016).

Previously, we reported that the long isoform produced from the *Eef1d* gene via alternative splicing is a transcription factor for heat-shock element (HSE)-containing genes (Kaitsuka et al., 2011). The short isoform produced from this gene, eEF1Bδ1, is a canonical translation elongation factor that forms an eEF1B complex with eEF1Bα, eEF1Bγ, and valyl-tRNA synthetase (ValRS), and functions as a guanine nucleotide exchange factor (GEF) for eEF1A (Le Sourd et al., 2006). This canonical eEF1Bδ1 is found in almost all metazoan species and is ubiquitously expressed in all tissues (Le Sourd et al., 2006). In contrast, the protein translated from the long isoform, eEF1BδL, is highly abundant in brain and testis by tissue-specific splicing (Kaitsuka et al., 2011). Of more interest, the expression of eEF1BδL is restricted to higher organisms, such as mammals and avians (Merkin et al., 2012; Kaitsuka and Matsushita, 2015). It appears that the function of this long isoform is needed for the advanced machinery of transcription/translation in the central nervous system. Stress-responsive gene regulation is important for proteostasis during normal development, and the loss of proteostasis can lead to several neurodegenerative diseases (Balch et al., 2008; Labbadia and Morimoto, 2015). Most of these genes are molecular chaperons, such as HSE-containing heat-shock proteins that are required for the maintenance of proteostasis (Kim et al., 2013).

The physiological role of eEF1BδL has not yet been elucidated. As mentioned above, it is known that the expression of this isoform is restricted to brain and testis, but the splicing regulators of this gene remain unknown. Recently, a truncated mutation in the exon specific to the long isoform of the human *EEF1D* gene (NM_001130053.1) was reported in a patient with a neurodevelopmental disorder (Reuter et al., 2017). The patient, who had a deletion of one nucleotide within the exon coding the N-terminal region of eEF1BδL, had severe intellectual disability, microcephaly, and a short stature (Reuter et al., 2017), indicating that eEF1BδL has a significant role in neural development. In this study, we generated *Eef1d* long isoform-specific knockout (KO) mice and investigated their phenotypes. Here, we show that these KO mice exhibited severe seizures in response to a loud sound. Surprisingly, the KO mice exposed to aversive stimuli during the fear conditioning test displayed repressed spontaneous activity in their home cage and on the other tasks regardless of seizure manifestation. Furthermore, a reduced brain size was observed in the KO mice which had been subjected to the fear conditioning test. These results suggested the physiological role of eEF1BδL as a buffer against excessive responses to challenging environmental stimuli. In our molecular study, we found that basal protein synthesis was augmented and endoplasmic reticulum (ER) stress markers were induced in the hippocampus of the KO mice, suggesting that these abnormalities could lead to their phenotypes as audiogenic seizures (AGS) and aversive stimuli-induced activity suppression.

## Materials and methods

### Generation of eEF1BδL-KO mice

A targeting vector was constructed to replace exon 3 of the *Eef1d* genomic locus with a DNA fragment containing a loxP-flanked frt-flanked PGK promoter, the neomycin resistance gene, and exon 3. A diphtheria toxin A fragment gene driven by the MC-1 promoter was introduced at the 5′ end of the targeting vector. The linearized targeting vector was introduced into HK3i embryonic stem (ES) cells (Kiyonari et al., 2010), and the cells were selected in G418. Genomic DNA from ES cell clones was screened by PCR. Homologous recombination was confirmed by Southern blot analysis using external 3′,5′ and neomycin probes. A correctly targeted ES clone was injected into 8-cell stage embryos from CD-1 mice. Chimeric mice were mated with C57BL/6 female mice for germinal transmission. Heterozygous mice (flox/+) (Accession No. CDB0816K: http://www2.clst.riken.jp/arg/index.html) were mated with CAG-FLPe mice (Kanki et al., 2006) to excise the frt-flanked neomycin cassette, and then mated with CAG-Cre mice (Matsumura et al., 2004) to excise the loxP-flanked exon 3. Both transgenic mouse strains (RBRC01834 and RBRC01828) were provided by RIKEN BRC through the National Bio-Resource Project of the MEXT, Japan. Heterozygous mice (Δ exon3/+) were backcrossed to C57BL/6J for at least seven generations. Homozygous mice (Δ exon3/Δ exon3) were obtained by intercrossing the heterozygous mice. PCR genotyping was performed with the following primers: Primer 1, ACTAGAAACATCAGCCACCTC; Primer 2, AGACAGCTGTCTCTAGACTGG; Primer 3, TCAGACATGAGCTCTTGGATC. PCR product sizes are 266, 341 or 583 base pairs for wild-type, floxed or knockout allele, respectively.

### Western blot analysis

Mouse brain tissues were homogenized in RIPA buffer (50 mM Tris-HCl, pH 7.5, 4 mM EGTA, 150 mM NaCl, 50 mM NaF, 1 mM Na_3_VO_4_, 30 mM Na_4_P_2_O_7_·10H_2_O, 10 mM EDTA, 1% triton X-100, 50 μg/ml aprotinin, 50 μg/ml leupeptin, 1 mM PMSF, 1 mM DTT). After sonication, the insoluble materials were removed by centrifugation at 15,000 rpm for 15 min. The supernatants were then mixed with Laemmli's sample buffer (0.38 M Tris-HCl, pH 6.8, 12% SDS, 30% β-mercaptoethanol, 10% glycerol, 0.05% bromophenol blue) and boiled for 4 min. The samples were then subjected to sodium dodecyl sulfate polyacrylamide gel electrophoresis (SDS-PAGE) and transferred onto polyvinylidene difluoride (PVDF) membranes. The membranes were incubated with following primary antibodies: anti-β-actin (Chemicon, Temecula, CA), anti-EEF1D, anti-EEF1B2, anti-EEF1A2 (Protein Tech Group, Chicago, IL) and anti-ATF-4 (Cell Signaling Technology, Danvers, MA). The membranes were subsequently incubated with HRP-conjugated secondary antibody (Cell Signaling Technology, Danvers, MA) and the immunoreactive proteins were visualized by Amersham ECL Prime (GE Healthcare, Buckinghamshire, England).

### qPCR analysis

Mice were subjected to hot environment at 38°C for 4 h in incubator camber as described previously with slight modifications (Sharma et al., 2010). The brain was then dissected and total RNA was extracted using TRIzol reagent (Thermo Fisher Scientific, Waltham, MA). cDNA was prepared by reverse transcription of 500 ng of total RNA using the SuperScript® VILO™ cDNA Synthesis Kit (Thermo Fisher Scientific). The resulting cDNAs were amplified using PowerUp™ SYBR® Green Master Mix (Thermo Fisher Scientific) and analyzed with a ViiA7 instrument (Thermo Fisher Scientific). All mRNA expression data were normalized to the *Actb* expression in a corresponding sample. The primer sequences are listed in Table 1.

**Table 1:** Primer list used in quantitative real-time PCR analysis.

**Organism**	**Genes**	**Sequences (forward and reverse)**
Mouse	*Act*	CCTCATGAAGATCCTGACCGA TTGCCAATAGTGATGACCTGG
Mouse	*Chop*	CCACCACACCTGAAAGCAGAA AGGTGAAAGGCAGGGACTCA
Mouse	*Atf4*	ATGGCCGGCTATGGATGAT CGAAGTCAAACTCTTTCAGATCCATT
Mouse	*Cryab*	ACACCGGACTCTCAGAGATGC TGGTGAGAGGATCCACATCGGC
Mouse	*Dnajb1*	AAGATCCTGACCATCGAAGTG AGTGCAACCACAGAGAGCCTC
Mouse	*Hmox1*	AGAATGCTGAGTTCATGAAG TGAGAGGTCACCCAGGTAGC
Mouse	*Hspa1a/b*	TGTCCATCCTGACGATCGACG TGGACGACAGCGTCCTCTTGG
Mouse	*Hspb1*	GTCTCGGAGATCCGACAGAC ACACCTGGAGGGAGCGTGTA
Mouse	*Hspb2*	ACTGCCGAGTACGAATTTGCC TTGCCTTCACTGAGCCTGAG

### Determination of the bulk translation rate

Primary cultured neurons were obtained from the hippocampus of fetal WT or KO mice on embryonic day 17 and maintained in neurobasal medium with 2% B-27 supplement (Thermo Fisher Scientific) at 37°C with 5% CO_2_. At 7 day, neurons were pre-incubated in methionine- and cysteine-free culture medium for 30 min. Next, ^35^S-methionine/cysteine (0.1 mCi/ml) was added to the medium and neurons were labeled for 30 min. Total lysates were prepared and subjected to SDS-PAGE, followed by autoradiography. SUnSET was performed as previously reported (Schmidt et al., 2009). Neurons were labeled with 5 μg/ml puromycin for 30 min. The neurons were lysed and subjected to western blot analysis with anti-puromycin antibody (Cosmobio, Tokyo, Japan).

### Experiments involving animals

All procedures involving mice were performed in compliance with the National Institutes of Health and RIKEN Kobe Animal Facility guidelines, and were approved by the Laboratory Animal Care and Use Committees of Kumamoto University and Institutional Animal Care and Use Committee of RIKEN Kobe Branch.

All animals were housed in 3–5 animals per cage (W14.5 × D34 × H14.5 cm) under a 12-h dark–light cycle (light from 07:00 to 19:00) at 22 ± 1°C and 40–80% of humidity with *ad libitum* food and water.

The open-field test was performed on male mice at the age of 8–30 weeks as described previously with slight modifications (Yamasaki et al., 2008). The test chamber was nearly cubic (W45 × D45 × H40 cm) and illuminated at 100 lx. The floor was divided into 81 equal squares, and the central 49 squares were defined as the center area. Each mouse was placed in a corner of the chamber, and behavior was recorded for 60 min using a video recorder. The distance traveled and time spent in the center area was analyzed by LimeLight software (version 4.0.01, Actimetrics, Wilmette, IL).

The elevated plus-maze test was performed on male mice at the age of 8–30 weeks as described previously with slight modifications (Komada et al., 2008). The maze consisted of two opposite-facing open arms (30 × 7 cm), two opposite-facing closed arms (30 × 7 × 20 cm), a central area measuring 6 × 6 cm, and was mounted on a base 37 cm above the floor. Each mouse was placed in the central area facing one of the closed arms and a 10-min test session was initiated. During the test, the movement of mouse was tracked and its trace was monitored by Limelight video camera based tracking system. Then the total distance traveled, the number of total entries, the percent of entries into the open arms, and the time spent in the open arms were analyzed by the LimeLight software (version 4.0.01, Actimetrics, Wilmette, IL).

The Y-maze test was performed on male mice at the age of 17–38 weeks as described previously with slight modifications (Tamada et al., 2010). The maze consisted of three arms (40 cm length each). The mouse was placed at the end of one arm and allowed to move freely through the maze during a 10-min session. The series of arm entries was recorded with a charge coupled device (CCD) camera connected to a computer. An alternation was defined as entries into all three arms on consecutive occasions.

The contextual and cued fear conditioning test was performed on mice at the age of 18–44 weeks as described previously with slight modifications (Miyakawa et al., 2003; Tamada et al., 2010). On the conditioning day, mice were placed in a test chamber (W26 × D34 × H29 cm) and allowed to explore the conditioning context for 2 min. Auditory cue of pure tone (90 dBA), which served as the conditioned stimuli (CS) was presented for 30 s and co-terminated with a constant current of 0.3-mA foot shock for 2 s, which served as unconditioned stimulus (US). This tone-shock (CS-US) pairing was repeated twice with an interval of 2 min. One day later, the conditioned mice were re-exposed to the conditioning chamber for 6 min without the auditory cue, and freezing was measured to test for contextual fear memory. After 2 h interval, the mice were tested in a novel chamber consisting of a triangle ceiling and white floor for 3 min, followed by the auditory cue for 3 min to test for cue-induced freezing. All data were analyzed using the FreezeFrame-3 Conditioned Fear System (ActiMetrics). Data were evaluated as a percentage of the time spent freezing per minute. Images were captured at 1 frame per second. The number of pixel changes based on the movement of a mouse between successive frames was measured. When this area was below a certain threshold (i.e., 20 pixels), the behavior was judged as “freezing.” When the amount of area equaled or exceeded the threshold, the behavior was considered as “non-freezing.” The optimal threshold (amount of pixels) to judge freezing was determined by adjusting it to the amount of freezing measured by human observation. “Immobility” that lasted less than the defined time threshold (i.e., 2 s) was not included in the analysis.

At 2 weeks after all mice had been subjected to the fear conditioning test, the treadmill test was performed as described previously with slight modifications (Ortiz-Abalia et al., 2008). The treadmill LE8710M (PanLab, Barcelona, Spain) consisted of a belt (24 cm long and 5 cm wide) that was run at a varying speed. At the end of the treadmill, there was an electrified grid that gave the mice a shock on the foot (0.2 mA) whenever they fell off the belt. The mice were placed on the top of the already-moving belt at the speed of 25 cm/s, facing away from the electrified grid and in the opposite direction to the travel of the belt. Then the mice were subjected to running for 10 min, and the number of electric shocks given to the mice were counted.

Six weeks after all mice had been subjected to the fear conditioning test, locomotor activity of those animals in their home cage was measured. The mice were acclimated to the single housing environment for 2 days, and locomotor activity data was then collected for 24 h with ACTIMO-100 (Shinfactory, Fukuoka, Japan). Total break number of infrared beams was counted every 5 min during dark and light phase, and the averaged counts per 5 min was shown as locomotor activity.

Seventeen weeks after all mice had been subjected to the fear conditioning test, audiogenic seizures (AGS) were assessed under identical experimental conditions as used for fear conditioning test without foot shock according to a previous report with modifications (Nosten-Bertrand et al., 2008). After 15 s of habituation, the animals were exposed to 2900 Hz pure tone at 90 dBA for 90 s. The intensity of the response pattern was rated blind to the genotypes: 0, no response; 1, wild running; 2, wild running and subsequent clonic seizures and/or tonic flexion and extension. Wild running is defined as uncontrollable, pronounced, undirected running and thrashing (Ross and Coleman, 2000).

### Statistical analyses

The data in all graphs are expressed as mean ± standard error of the mean (SEM). Statistical analyses were performed by two-tailed unpaired Student's *t*-test or two-way repeated measures analysis of variance (ANOVA). To exclude potential type I errors, the false discovery rate (FDR) was calculated on multiple comparisons of the qPCR, western blot, translation rate analysis, body/brain weight and behavioral analysis performed before or after the fear conditioning test as described previously (Benjamini and Hochberg, 1995; Benjamini et al., 2008), and the FDR-adjusted significance level was applied to each analysis.

## Results

### Generation of eEF1BδL-KO mice

**Figure 1:**
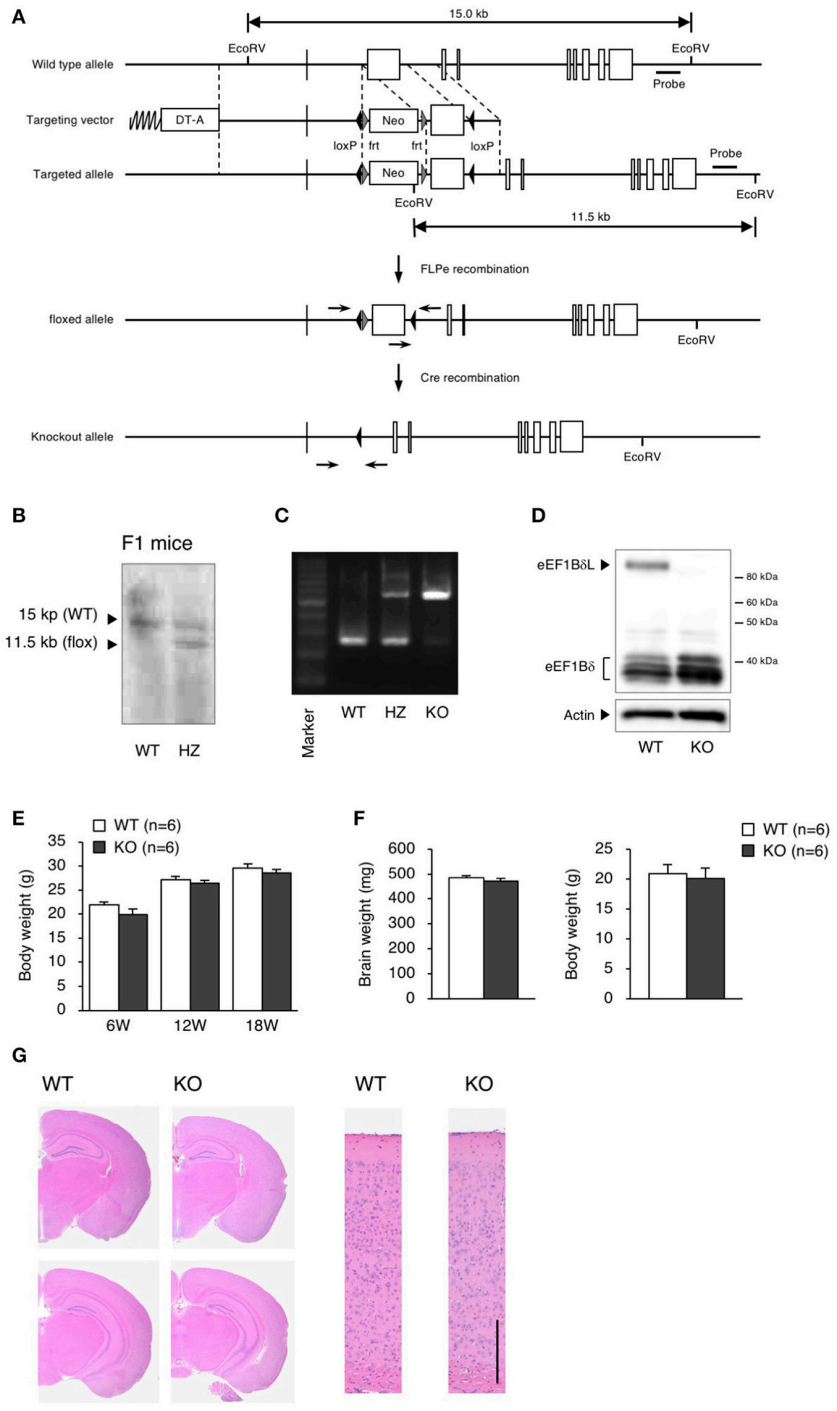
Generation of eEF1BδL-KO mice. **(A)** Schematic diagram of the endogenous *Eef1d* locus and the targeting vector used to engineer embryonic stem (ES) cells by homologous recombination. The lengths of the genomic fragments indicated are the lengths when digested by EcoRV. The 3′ probe was used for Southern blotting to confirm homologous recombination. DT-A, diphtheria toxin-A; neo, neomycin-resistant gene. **(B)** Southern blot analysis of genomic DNA from F1 mice using the 3′ probe after digestion with EcoRV. **(C)** PCR genotyping of genomic DNA from wild-type (WT), heterozygous (HZ) and knockout (KO) mice using primers crossing loxP. **(D)** Western blot analysis of brain lysates from WT and KO mice. Actin was used as a loading control. **(E)** Body weight of WT and KO male mice at the age of 6, 12, and 18 weeks. *n* = 6 for each. **(F)** Brain weight of WT and KO male mice at the age of 5–13 weeks. *n* = 6 (WT mice) or 8 (KO mice). **(G)** Hematoxylin and eosin (H&E) staining of brain sections from WT and KO male mice sacrificed at the age of 16 weeks. Left panel: coronal section. Right panel: cortical layer. Scale bar: 200 μm.

**Figure 2:**
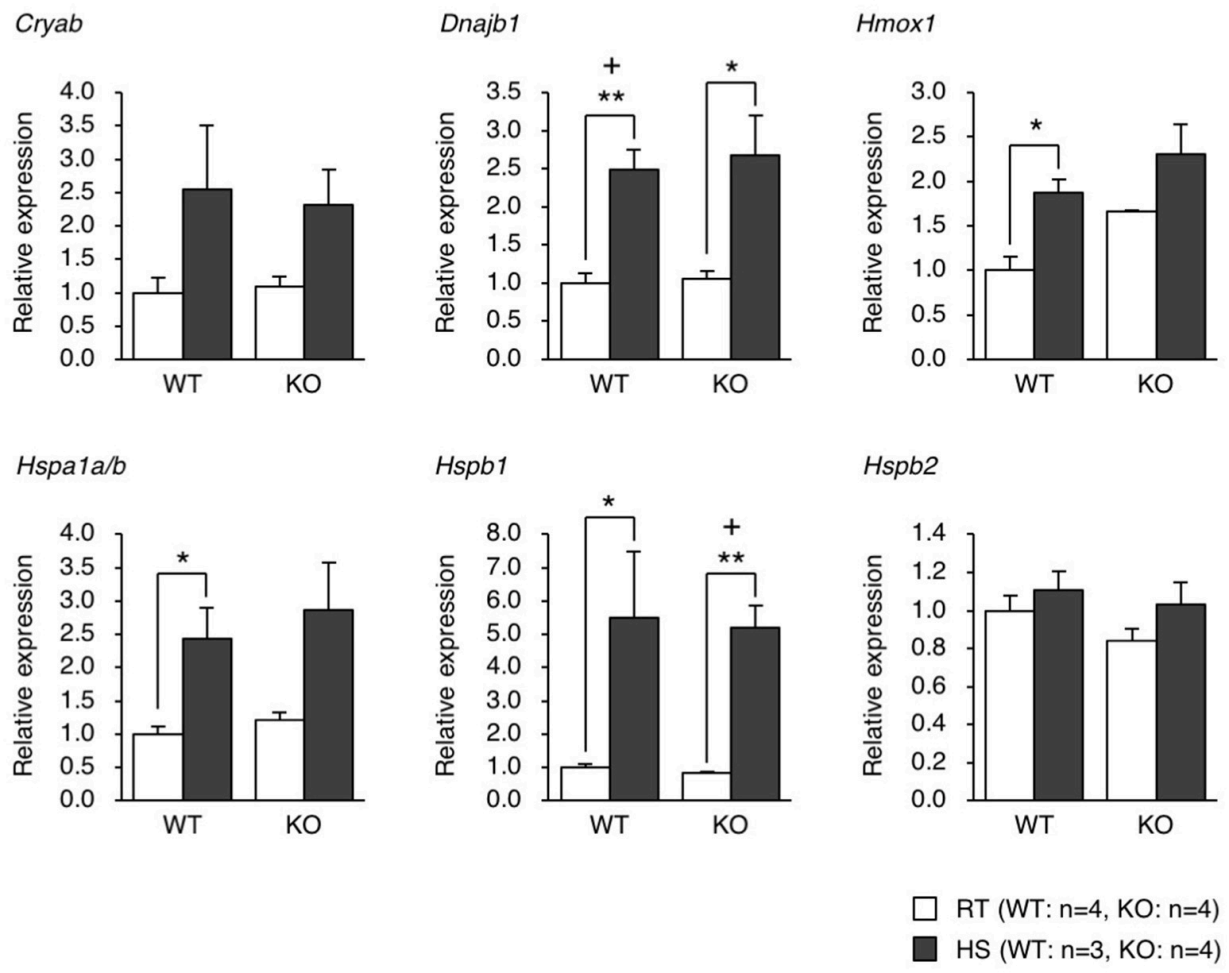
qPCR analysis of the gene expression of heat shock proteins in the brain of mice exposed to heat. WT and KO mice were exposed to a hot environment (38°C) for 4 h. The brain was then dissected, and the total RNA was extracted and subjected to qPCR analysis. ^*^p < 0.05, ^**^p < 0.01, *n* = 3 or 4, Student's *t*-test. (+) denotes significant differences after adjustment by false discovery rate (FDR) analysis.

We generated eEF1BδL-KO mice using the standard protocol shown in Figure 1A. Homologous recombination was confirmed by Southern blot analysis using tail DNA from the founder 1 (F1) mice (Figure 1B). Genotyping was performed by PCR using primers crossing the flox region on pups form heterozygous female mice mated with heterozygous male mice (Figure 1C). To confirm the selective deletion of eEF1BδL, the brain lysates from wild-type (WT) and KO mice were subjected to western blot analysis. The eEF1BδL protein was completely abolished in the KO mice, while the eEF1Bδ1 protein remained (Figure 1D). After birth, KO mice developed normally and gained weight similar to that of WT mice (Figure 1E). Although the expression of eEF1BδL was also detected in the testis (Kaitsuka et al., 2011), both male and female KO mice were fertile, despite the deletion of eEF1BδL from their testes. The weight and morphology of the brain were normal in naïve KO mice (Figures 1F,G). Next, we tested for the expression of HSE-containing genes in the brain of mice exposed to a hot environment. Hyperthermia induces HSE-containing genes including heat shock proteins (Westman and Sharma, 1998). When the animals are exposed to hot environment above the normal body temperature, hyperthermia and heat shock proteins are induced in several tissues especially brain (Sharma et al., 2010). Contrary to our previous report, no significant differences were observed between the WT and KO mice with or without exposure to hot environment (Student's *t*-test, Dnajb1 RT vs. HS: WT *p* = 0.0028, KO *p* = 0.0210, Hmox1 RT vs. HS: WT *p* = 0.0104, Hspa1a/b RT vs. HS: WT *p* = 0.0193, Hspb1 RT vs. HS: WT *p* = 0.0406, KO *p* = 0.0005; Figure 2). The FDR-adjusted significant level (*p* < 0.0063) indicated that the differences in Dnajb1 RT vs. HS (WT) and Hspb1 RT vs. HS (KO) were still significant.

### eEF1BδL-KO mice showed normal behavior before the fear conditioning test

**Figure 3:**
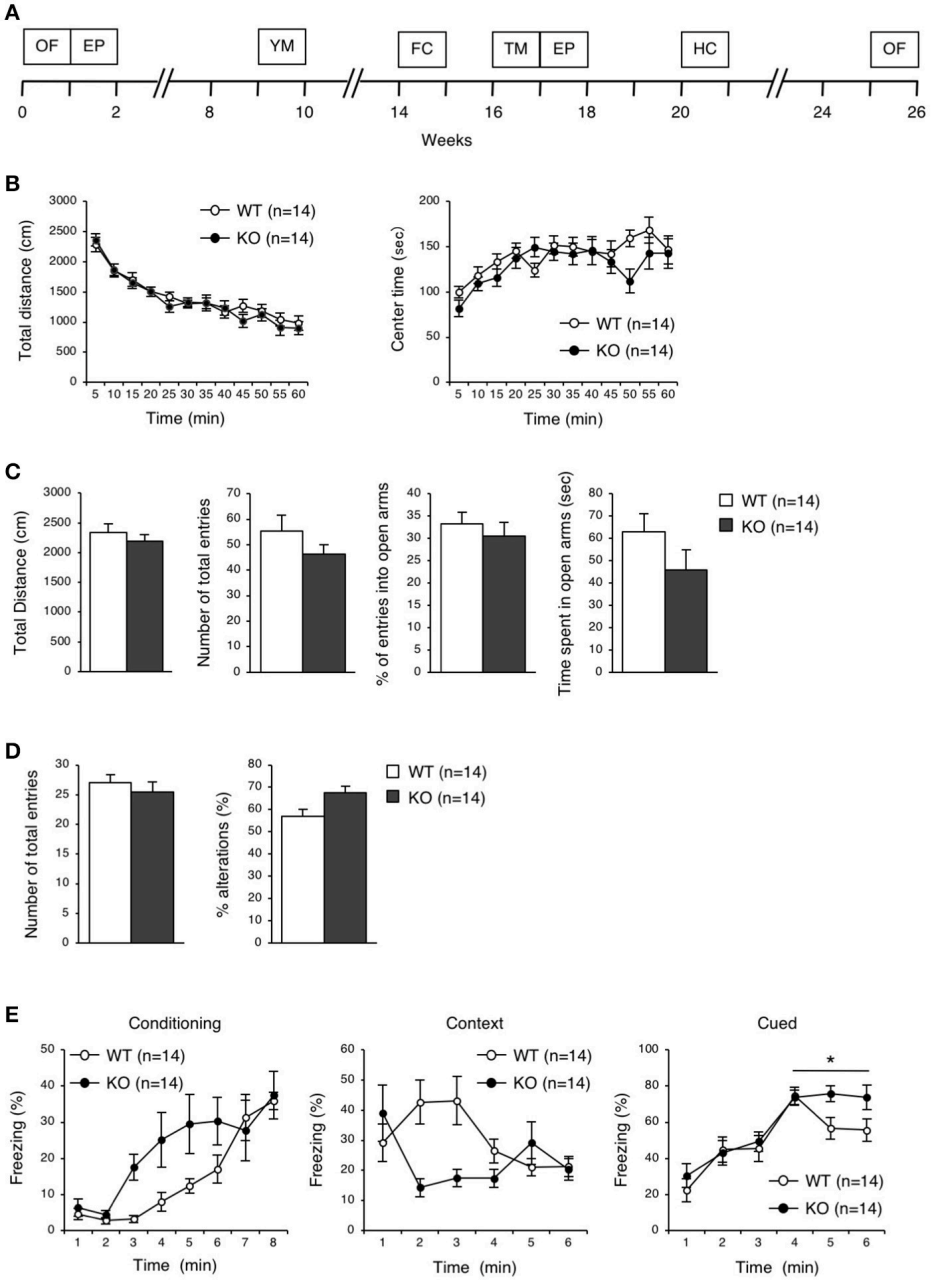
Behavioral analysis of eEF1BδL-KO mice before the fear conditioning test. **(A)** A timeline of the behavioral tests which were performed on WT and KO mice. 0 week corresponds to the beginning of open-field test. OF, open-field; EP, elevated plus; YM, Y-maze; FC, fear conditioning; TM, treadmill; HC, home cage. **(B)** Open-field test on WT and KO male mice at the age of 8–30 weeks. The total distance traveled and time spent in the center area were measured for 60 min, and the data collected at every 5 min are displayed. *n* = 14 for each. **(C)** Elevated plus-maze test on WT and KO male mice at the age of 8–30 weeks. The total distance traveled, number of total entries, percent of entries into the open arms, and time spent in the open arms were measured. *n* = 14 for each. **(D)** Y-maze test on WT and KO male mice at the age of 17–38 weeks. The number of total entries and percent of alternations were measured. *n* = 14 for each. **(E)** Fear conditioning test on WT and KO male mice at the age of 18–44 weeks. The percent of time spent freezing was measured at conditioning, context and cued testing. ^*^p < 0.05, *n* = 14 for each, two-way repeated measures ANOVA.

To elucidate the behavioral phenotypes of eEF1BδL-KO mice, we conducted various behavioral analyses on both WT and KO male mice (Figure 3A). Spontaneous locomotor activity and anxiety-related behavior were evaluated by the open-field test, anxiety-related behavior was evaluated on the elevated plus maze, and spatial working memory was evaluated by Y-maze test. There were no significant differences between the WT and KO mice in the open-field test, elevated plus-maze test, or Y-maze test (Figures 3B–D).

### eEF1BδL-KO mice exhibited audiogenic seizures

To examine the ability for fear learning, we conducted a fear conditioning test on WT and KO mice (Figure 3E). Surprisingly, several KO mice exhibited tonic-chronic seizures in response to stimulation consisting of foot shock and an auditory cue at the conditioning trial (Supplemental Movies S1, S2). As a result, five of the 14 mice in the KO group showed a score of 2 (wild running and the subsequent clonic seizures and/or tonic flexion and extension) (Average score, WT: 0 ± 0, KO: 0.71 ± 0.27, Student's *t*-test, *p* = 0.0124; Table 2). Overall, slight differences were observed in a percent of freezing in cued test between WT and KO mice, while not in a conditioning and contextual test (two-way repeated measures ANOVA, Conditioning, *p* = 0.07955; Context, *p* = 0.13245; Cued, *p* = 0.04387; Figure 3E). We next tested whether these mice would experience seizures in response to either foot shock or auditory cues. Three of 11 KO mice showed a score of 2 only for auditory cues, demonstrating that the KO mice exhibited audiogenic seizures (AGS) (Average score, WT: 0 ± 0, KO: 0.82 ± 0.26, Student's *t*-test, *p* = 0.0018; Table 2), and that the eEF1BδL-KO mice had an increased susceptibility to AGS. The FDR-adjusted significant level (*p* < 0.02) indicated that the differences in average seizure score at FC and AGS test were still significant.

**Table 2:** Seizure score from the fear conditioning test and audiogenic seizure test.

				**Response**		
**Task**	**Genotype**	**Sex**	***n***	**0**	**1**	**2**
FC	WT	M	14	14 (100)	0 (0)	0 (0)
FC	KO	M	14	9 (64.3)	0 (0)	5 (35.7)
AGS	WT	M	14	14 (100)	0 (0)	0 (0)
AGS	KO	M	11	5 (45.5)	3 (27.3)	3 (27.3)

*0, no response; 1, wild running; 2, wild running and the subsequent clonic seizures and/or tonic flexion and extension; FC, fear conditioning; AGS, audiogenic seizure; WT, wild-type; KO, knockout; M, male. The values in parenthesis are a percentage of animals which exhibited each response*.

### Compensatory increase in canonical eEF1Bδ1

**Figure 4:**
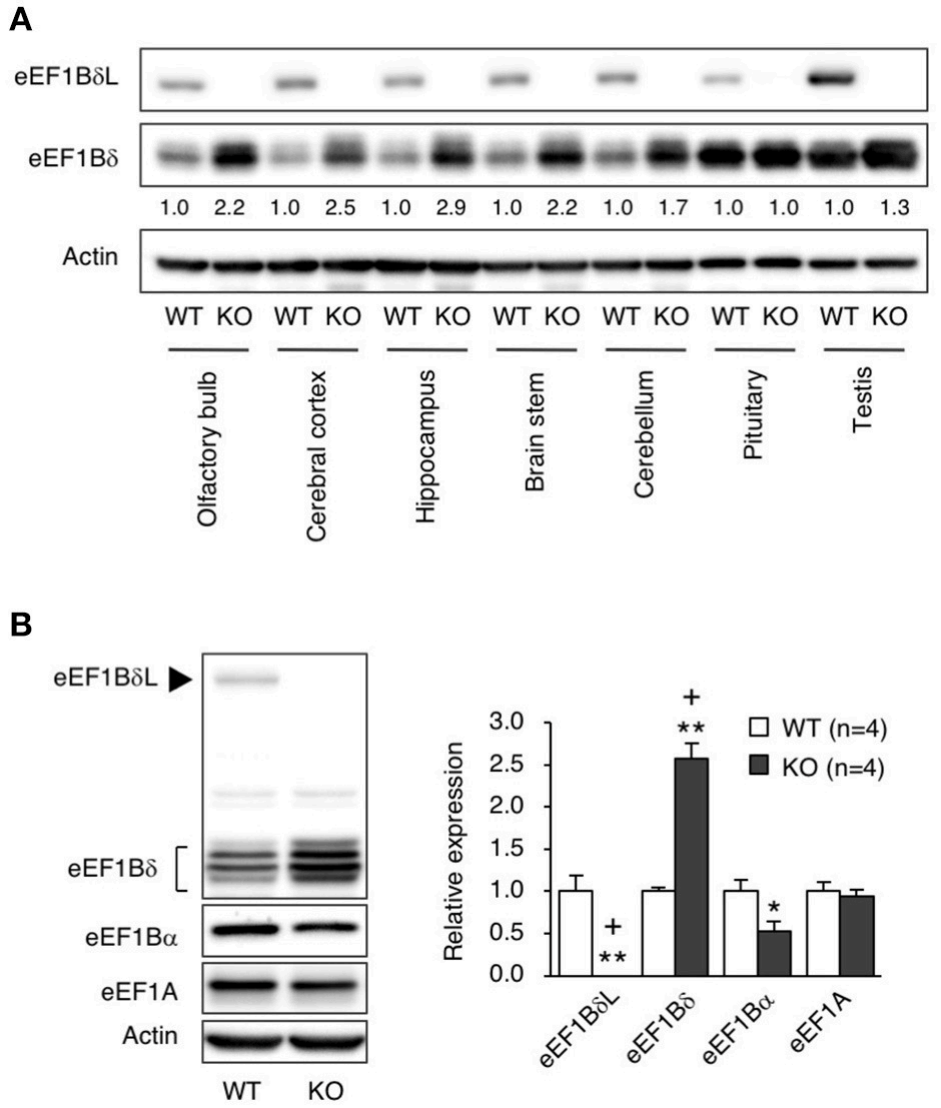
The levels of elongation factor family proteins in eEF1BδL-KO mice. **(A)** Western blot analysis of the long and short isoforms of eEF1Bδ in several brain regions from WT and KO male mice sacrificed at the age of 5 weeks. Actin was used as a loading control. **(B)** Western blot analysis of eEF1A, eEF1Bα, eEF1Bδ, and eEF1BδL in the hippocampal tissues from WT and KO male mice sacrificed at the age of 4–8 weeks. Actin was used as a loading control. Left panel: representative blot image. Actin was used as a loading control. Right panel: graph showing the quantitative data. ^*^p < 0.05, ^**^p < 0.01, *n* = 4 for each, Student's *t*-test. (+) denotes significant differences after adjustment by FDR analysis.

To elucidate which brain regions express eEF1BδL, we measured the levels of eEF1BδL in dissected regions of the brain, including the olfactory bulb, cerebral cortex, hippocampus, brain stem, cerebellum, and pituitary. In WT mice, all regions expressed similar levels of eEF1BδL. In contrast, in KO mice, none of the regions showed eEF1BδL expression, but eEF1Bδ1, the short isoform of *Eef1d*, was found at increased levels in the olfactory bulb, cerebral cortex, hippocampus, brain stem, and cerebellum (Figure 4A). We next examined and quantified the protein levels, including those of other elongation factor family members in the hippocampus. In KO mice, the level of eEF1Bδ1 was significantly increased, while the level of eEF1Bα was decreased (Student's *t*-test, eEF1BδL: *p* = 0.0017, eEF1Bδ: *p* = 0.0002, eEF1Bα: *p* = 0.0380; Figure 4B), showing that the balance between the levels of elongation factor family members was disrupted in the KO mice. The FDR-adjusted significant level (*p* < 0.025) indicated that the differences in eEF1BδL and eEF1Bδ levels were still significant.

### Suppressed spontaneous locomotor activity in eEF1BδL-KO mice after fear conditioning

**Figure 5:**
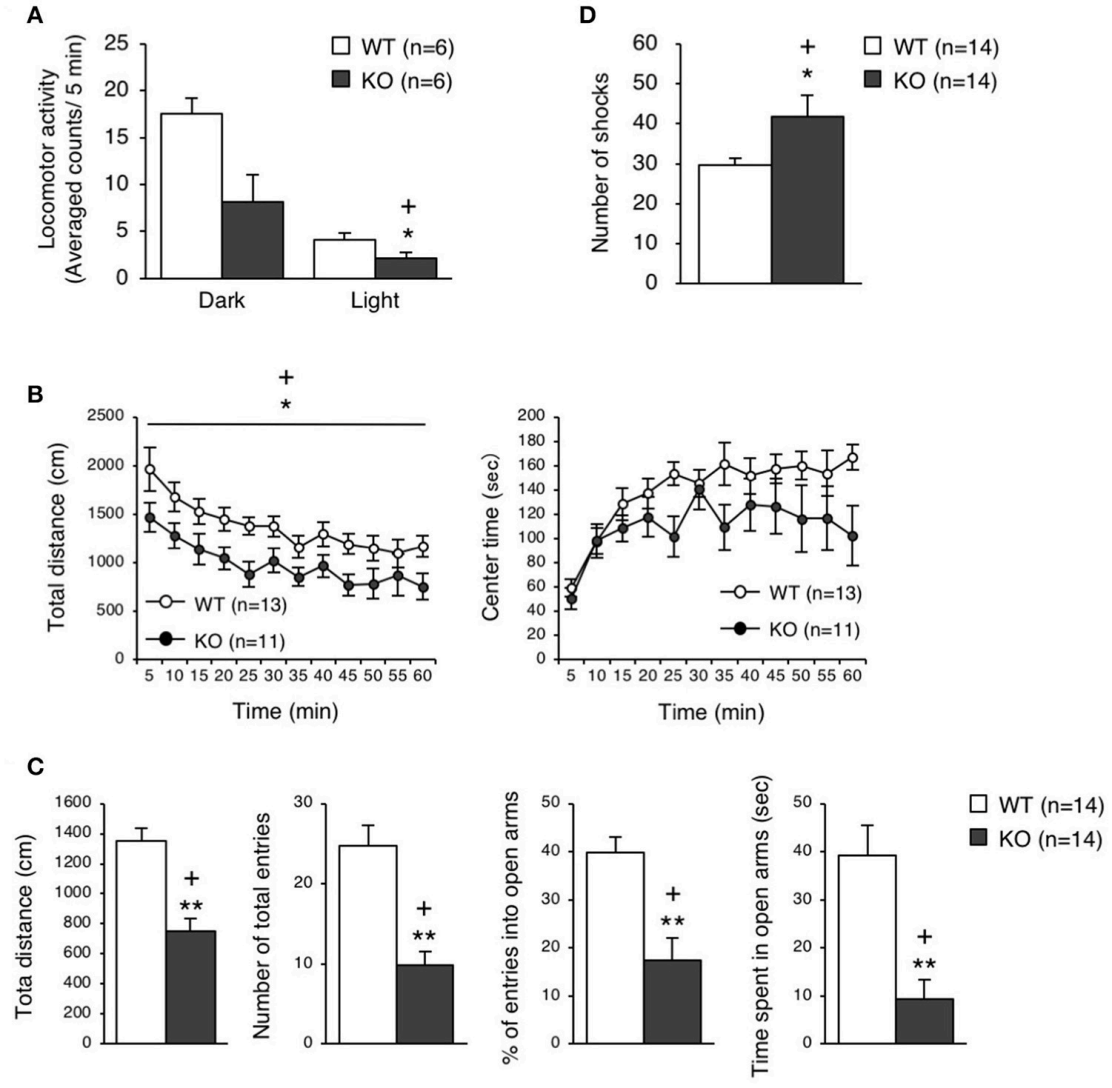
Behavioral analysis of eEF1BδL-KO mice after the fear conditioning test. **(A)** Spontaneous locomotor activity in the home cage 6 weeks after the fear conditioning test. Locomotor activity was measured during the dark and light phases. ^*^p < 0.05, *n* = 6 for each, Student's *t*-test. (+) denotes significant differences after adjustment by FDR analysis. **(B)** Open-field test on WT and KO male mice 11 weeks after fear conditioning. The total distance traveled and time spent in the center area were measured for 60 min, and the data obtained at 5-min intervals are displayed. ^*^p < 0.05, *n* = 13 (WT mice) or 11 (KO mice), two-way repeated measures ANOVA. (+) denotes significant differences after adjustment by FDR analysis. **(C)** Elevated plus-maze test on WT and KO male mice 3 weeks after fear conditioning. The total distance traveled, number of total entries, percent of entries into the open arms, and time spent in the open arms were measured. ^**^p < 0.01, *n* = 14 for each, Student's *t*-test. (+) denotes significant differences after adjustment by FDR analysis. **(D)** Treadmill test on WT and KO male mice 2 weeks after fear conditioning. The number of electric shock that the mice received was counted. ^*^p < 0.05, *n* = 14 for each, Student's *t*-test. (+) denotes significant differences after adjustment by FDR analysis.

The eEF1BδL-KO mice, which had been subjected to the fear conditioning test, appeared to be subdued as less active. Therefore, we examined their spontaneous locomotor activity in their home cage 6 weeks after the fear conditioning test had been conducted. Surprisingly, the KO mice showed lower activity when compared to the WT mice regardless of whether they were epileptic or non-epileptic animals (Student's *t*-test, Dark: *p* = 0.0184, Light: *p* = 0.0561; Figure 5A). Furthermore, at 11 or 3 weeks after the fear conditioning test have been conducted, the KO mice were also less active than the WT mice in the open-field test (two-way repeated measures ANOVA, total distance: *p* = 0.0225, center time: *p* = 0.1803; Figure 5B) and elevated plus-maze test (Student's *t*-test, total distance: *p* = 0.00004, number of total entries: *p* = 0.00007, % of entries into open arms: *p* = 0.00062, time spent in open arms: *p* = 0.00049; Figure 5C). This result suggests that the auditory cues and foot shocks given during the fear conditioning test had some deleterious action on the KO mice. To determine whether the motor function was affected in the KO mice, we conducted the treadmill test and found that the running ability of KO mice was significantly worse than WT mice, as revealed by the higher number of shocks received (Student's *t*-test, *p* = 0.039; Figure 5D). The FDR-adjusted significant level (*p* < 0.0389) indicated that all differences in behavioral analysis performed after the fear conditioning test were still significant.

### Brain atrophy in eEF1BδL-KO mice

**Figure 6:**
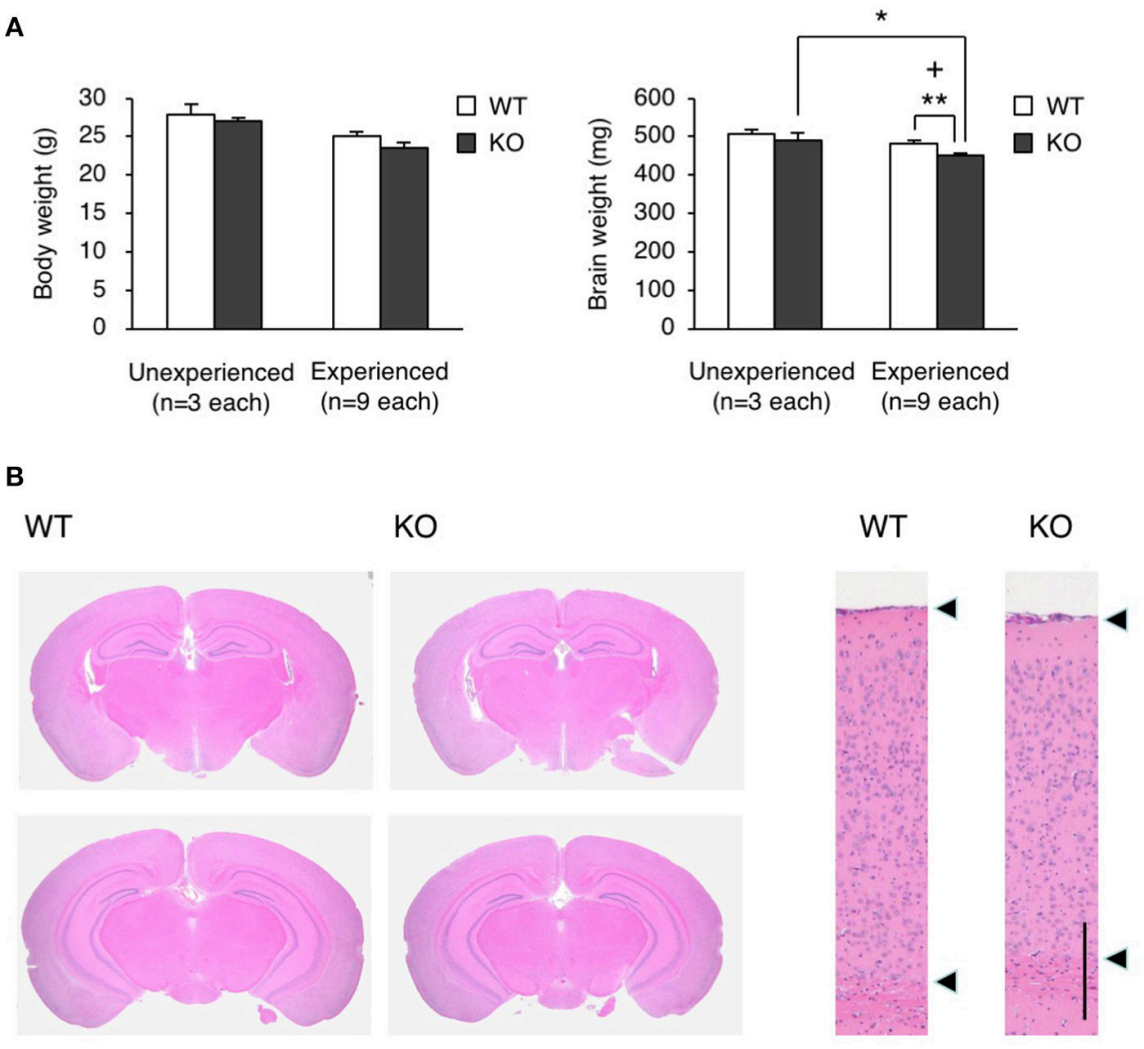
Brain atrophy in eEF1BδL-KO mice. **(A)** The brain weight and body weight of WT and KO male mice sacrificed at the age of 8–13 weeks. Mice were subjected to the fear conditioning test 2 weeks before they were sacrificed. Unexperienced, mice with no experience of fear conditioning test; Experienced, mice with experience of fear conditioning test. ^*^p < 0.05, ^**^p < 0.01, *n* = 9 (WT mice) or 9 (KO mice), Student's *t*-test. (+) denotes significant differences after adjustment by FDR analysis. **(B)** Hematoxylin and eosin (H&E) staining of the brain sections from WT and KO male mice sacrificed at the age of 12 weeks. Mice were subjected to the fear conditioning test 2 weeks before they were sacrificed. Left panel: coronal section. Right panel: cortical layer. Scale bar: 200 μm.

To clarify how the experience during the fear conditioning test induced the restraint in locomotor activity, we measured the brain weight of WT and KO mice 2 weeks after the test, and found that the brain weight of KO mice was significantly less than that of WT mice in this experienced group (Student's *t*-test, *p* = 0.0057), while there was no significant difference between WT and KO mice in unexperienced group as with no experience of fear conditioning test (Student's *t*-test, *p* = 0.4950; Figure 6A). Notably, the experience of the fear conditioning test reduced the brain weight in KO mice but not in WT mice (Student's *t*-test, brain weight, experienced vs. unexperienced: WT *p* = 0.1397, KO *p* = 0.0344; body weight, experienced vs. unexperienced: WT *p* = 0.0512, KO *p* = 0.0533; Figure 6A). We also performed hematoxylin and eosin (H&E) staining on the WT and KO brain samples, and found atrophy of the hippocampus and midbrain, and a reduced thickness of the cortical layer (Figure 6B). The FDR-adjusted significant level (*p* < 0.0125) indicated that the differences in brain weight between WT and KO mice of experienced group were still significant.

### Augmented basal protein synthesis in eEF1BδL-KO mice

**Figure 7:**
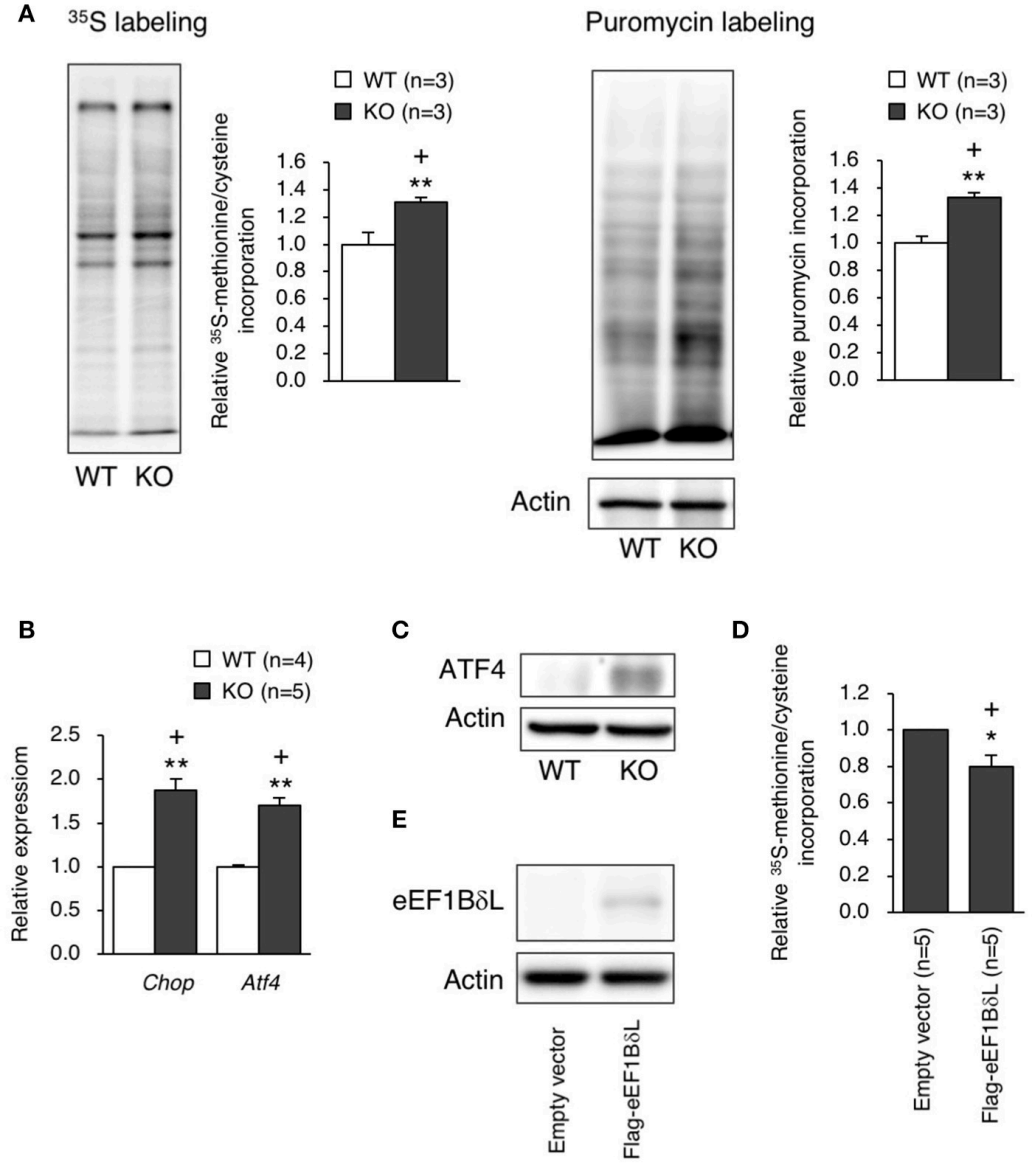
Protein synthesis and ER stress markers in eEF1BδL-KO mice. **(A)** The rate of protein translation was measured in cultured hippocampal neurons from WT and KO mice. Left panel: representative autoradiography of ^35^S-methionine/cysteine labeling and a graph showing the quantitative data. ^**^p < 0.01, *n* = 3 for each, Student's *t*-test. Right panel: representative image of a blot with puromycin labeling and a graph showing the quantitative data. ^**^p < 0.01, *n* = 3 for each, Student's *t*-test. Actin was used as a loading control. (+) denotes significant differences after adjustment by FDR analysis. **(B)** qPCR was performed for ER stress markers using total RNA isolated from the hippocampal tissues of WT and KO mice. ^**^p < 0.01, *n* = 4 (WT mice) or 5 (KO mice), Student's *t*-test. (+) denotes significant differences after adjustment by FDR analysis. **(C)** Western blot analysis for ATF4 in the hippocampal tissues from WT and KO mice. Actin was used as a loading control. **(D)** HEK293 cells were transfected with empty vector or a plasmid expressing eEF1BδL. Twenty-four hours after transfection, the rate of protein translation was measured by ^35^S-methionine/cysteine labeling, followed by liquid scintillation counting. ^*^p < 0.05, *n* = 5 for each, Student's *t*-test. (+) denotes significant differences after adjustment by FDR analysis. **(E)** Western blot analysis for eEF1BδL in HEK293 cells transfected with empty vector or a plasmid expressing eEF1BδL. Actin was used as a loading control.

Because the balance between the levels of the elongation factor family members was disrupted in KO mice, we examined the protein translation efficiency in hippocampal neurons. The protein translation rate was significantly increased in the neurons of KO mice, which was confirmed by both ^35^S-methionine/cysteine labeling and the surface sensing of translation (SUnSET) method (Student's *t*-test, ^35^S labeling: *p* = 0.0043, puromycin labeling: *p* = 0.0046; Figure 7A). When excessive protein synthesis occurs, endoplasmic reticulum (ER) stress could be triggered (Scheper et al., 2007). Therefore, we measured ER stress markers, *Chop* and *Atf4*, in the hippocampus and found that the expression levels of *Chop* and *Atf4* were significantly higher (Student's *t*-test, *Chop*: *p* = 0.0075, *Atf4*: *p* = 0.0098; Figure 7B), along with an increased ATF4 protein level (Figure 7C), in the KO mice than in the WT mice. To test whether eEF1BδL has an inhibitory effect on protein translation, HEK293 cells were transfected with a plasmid expressing Flag-tagged eEF1BδL, and the translation rate was measured. The rate was significantly decreased in eEF1BδL-expressing cells, showing that eEF1BδL could have an inhibitory effect on protein synthesis (Student's *t*-test, *p* = 0.0363; Figures 7D,E). The FDR-adjusted significant level (*p* < 0.05) indicated that all differences were still significant.

## Discussion

In this study, we generated eEF1BδL-KO mice by selective deletion of the exon specific to the long isoform produced from the *Eef1d* gene. These KO mice showed normal behavior before the fear conditioning test. However, fear conditioning led to repressed behavior, as was seen by the decreased spontaneous locomotor activity in these mice. Furthermore, KO mice exhibited increased susceptibility to AGS.

### Deletion of eEF1BδL causes audiogenic seizures (AGS)

Some eEF1BδL-KO mice showed tonic-clonic seizures in response to a 90-dB loud sound (auditory cue at fear conditioning). Surprisingly, several KO mice died after exposure to only the loud sound due to seizure followed by suffocation. This type of seizure, AGS, is also the representative phenotype of *Fmr1*-KO mice, a model of fragile X syndrome (Musumeci et al., 2000; Chen and Toth, 2001; Yan et al., 2004). In *Fmr1*-KO mice, the basal protein synthesis level is increased, and this excessive protein synthesis is thought to be the primary cause of AGS (Dölen et al., 2007; Osterweil et al., 2013). Similar to the *Fmr1*-KO mice, eEF1BδL-KO mice showed increased basal protein synthesis levels (Figure 7A), suggesting that this abnormality might be the primary cause of AGS, like in *Fmr1*-KO mice.

### Deletion of eEF1BδL results in suppressed spontaneous locomotor activity after the exposure to aversive stimuli

The eEF1BδL-KO mice, which have been subjected to the fear conditioning test, showed remarkably repressed behavior. These experienced KO mice were less active in their home cage, and in the open-field test and elevated plus-maze test. Furthermore, the anxiety-related behaviors were enhanced in these experienced KO mice, as evaluated by the time spent in the open arms in the elevated plus maze. The fear conditioning test is thought to act as a stressor that induces behavioral changes via increased anxiety levels like post-traumatic stress disorder (PTSD) symptoms (Rau et al., 2005; Tronel and Alberini, 2007). Moreover, it has been reported that this test in combination with a strong intensity (1.0 mA) of foot shock caused a decrease in locomotor activity and lead to hippocampal apoptosis (Wang et al., 2012). In our study, despite the mild intensity (0.3 mA) of foot shock used, the sound and foot shock stimuli appeared to only have a deleterious effect in KO mice. Supporting this idea, brain atrophy was found in KO mice experienced of the task but not in naïve animals (Figure 6), indicating that these mice were weaker to such external stimuli. However, we cannot conclude on whether the reduced locomotor activity and the brain atrophy was caused from exposure to tone, foot shock or both stimuli. Furthermore, although only 36% of KO mice exhibited seizures at the conditioning trial of fear conditioning test, almost all mice showed suppressed locomotor activity after experience of this test. However, we still cannot explain why such difference occurred. Further experiments are needed for final conclusion.

### Deletion of eEF1BδL causes excess protein synthesis in hippocampal neurons

The basal protein synthesis level was increased in the hippocampal neurons of the KO mice (Figure 7A). One possibility regarding excessive protein synthesis in KO mice is that eEF1BδL might have a repressive effect on protein translation, as the overexpression of eEF1BδL had an inhibitory effect on basal protein synthesis (Figure 7D). eEF1BδL has a nuclear localization signal in the N-terminal region and localizes to the nucleus (Kaitsuka et al., 2011), indicating that this protein may change the subcellular localization of its binding partners eEF1A, eEF1Bγ, and ValRS. These changes might have an effect on translation efficiency. However, further investigations, including those to identify the types of proteins that are increased in KO mice, are needed to conclusively determine the effects on translation efficiency.

### Deletion of eEF1BδL causes induction of ER stress markers in hippocampus

Increased levels of unfolded proteins lead to an unfolded protein response (UPR), which enables the maintenance of protein-folding homeostasis (Schröder and Kaufman, 2005; Korennykh and Walter, 2012). However, when excessive protein synthesis occurs, UPR cannot relieve ER stress (Scheper et al., 2007). During ER stress, ATF4 protein is specifically translated, and the downstream pro-apoptotic gene *Chop* is induced (Harding et al., 2003; Marciniak et al., 2004; Frakes and Dillin, 2017). In eEF1BδL-KO mice, the levels of both ATF4 and Chop were increased, suggesting that the induced Chop could lead to the apoptosis of neurons. This might explain how the brain atrophy occurred in the KO mice. However, further investigation is needed for final conclusion on this mechanism.

### Implication of human *EEF1D* gene in psychiatric disorder

It has been reported that the long isoform of the human *EEF1D* gene (NM_001130053.1) is a candidate gene leading to neurodevelopmental disorders, such as intellectual disabilities (Reuter et al., 2017). A mutation in the *EEF1D* gene was found in a patient with a severe intellectual disability, microcephaly, and a short stature (Reuter et al., 2017); the mutation was a deletion in cDNA 69 that caused an amino acid change, Glu24 to Ser, followed by a translational stop codon 26 amino acids further due to the frame shift. This mutation might abolish the expression of only eEF1BδL, but not the canonical eEF1Bδ1, as was seen in the KO mice in our study. Of note, microcephaly is a phenotype similar to the brain atrophy observed in our eEF1BδL-KO mice, showing the phenocopy between human and mouse.

In conclusion, our results suggest that the brain-specific splicing isoform eEF1BδL has a physiological role for consequences of exposure to challenging environmental stimuli, such as loud sounds and foot shock. Moreover, the expression of eEF1BδL seems to act as translational repressor in neurons. Our study provides important insight for pathophysiology of psychiatric disorders, especially for patients carrying an *EEF1D* mutation.

## Author contributions

TK and MM conceived this study and designed experiments. TK performed most of the experiments. HK and AS supported the generation of knockout mice. KT provided critical advice. TK and MM wrote the paper.

### Conflict of interest statement

The authors declare that the research was conducted in the absence of any commercial or financial relationships that could be construed as a potential conflict of interest.

## Supplementary material

Movie S1 Representative movie of a WT mouse during the fear conditioning test. The movie was recorded during the first stimuli (tone and shock) at conditioning. The WT mouse responded normally to tone and jumped when shock was delivered.

Movie S2 Representative movie of a KO mouse during the fear conditioning test. The movie was recorded during the first stimuli (tone and shock) at conditioning. The KO mouse ran about when tone was delivered, and then displayed tonic-clonic seizure.
